# Systematic analysis of MASP-1 serves as a novel immune-related biomarker in sepsis and trauma followed by preliminary experimental validation

**DOI:** 10.3389/fmed.2024.1320811

**Published:** 2024-02-07

**Authors:** Lina Xian, Shaowen Cheng, Wei Chen, Changhui Zhong, Zhihua Hu, Xiaoyan Deng

**Affiliations:** ^1^Department of Intensive Care Unit, The First Affiliated Hospital of Hainan Medical University, Haikou, Hainan, China; ^2^Department of Wound Repair, The First Affiliated Hospital of Hainan Medical University, Haikou, Hainan, China

**Keywords:** MASP-1, sepsis, trauma, traumatic sepsis, CD8 T cells, IL6/JAK/STAT3 signaling

## Abstract

**Background:**

Dysregulated immune response in trauma and sepsis leads to the abnormal activation of the complement and coagulation systems. Mannose-binding lectin (MBL)-associated serine protease-1 (MASP-1) activates the lectin pathway of the complement system and mediates proinflammatory and procoagulant reactions. However, the potential effects of MASP-1 in trauma and sepsis have not yet been explored.

**Methods:**

We obtained five sepsis, two trauma, and one sepsis and trauma RNA-sequencing dataset from the Gene Expression Omnibus (GEO) database and conducted a comprehensive evaluation of the expression pattern, biological functions, and diagnostic value of MASP-1 in trauma and sepsis. Additionally, we investigated the association between MASP-1 expression and clinicopathological characteristics of trauma and sepsis. Furthermore, we collected clinical specimens to preliminarily validate the expression level and diagnostic efficacy of MASP-1 as well as the correlation of MASP-1 with clinical features of trauma and sepsis. Subsequently, we conducted a correlation analysis among MASP-1, immune cell infiltration, and immune and molecular pathways. Finally, we mechanistically analyzed the relationship among MASP-1, specific immune cells, and pivotal molecular pathways.

**Results:**

*MASP-1* expression was significantly upregulated in the trauma/sepsis samples compared to the control samples in the GEO datasets. MASP-1 exhibited excellent diagnostic values (AUC > 0.7) in multiple datasets and at multiple time points and could efficiently distinguish trauma/sepsis samples from the control samples. Moreover, *MASP-1* expression was significantly positively correlated with the severity of the disease (APACHE-II, CRP, and neutrophil levels). These results were further validated by real-time quantitative polymerase chain reaction and enzyme-linked immunosorbent assay. Functional enrichment analysis revealed that MASP-1 primarily promotes trauma and sepsis via the immune-related signaling pathway. MASP-1 was significantly correlated with the infiltration of specific immune cells (such as B cells, CD8 T cells, neutrophils, macrophages, and infiltrating lymphocytes) and immune and molecular pathways (such as checkpoint, HLA, IL6/JAK/STAT3 signaling, necrosis, T-cell co-inhibition, and T-cell co-stimulation). Finally, analysis of the transcription and single-cell data revealed that MASP-1 was specifically expressed in T cells, and further correlation analysis revealed a close correlation between MASP-1 expression, proportion of CD8 T cells, and IL6/JAK/STAT3 signaling scores.

**Conclusion:**

Our results suggest that MASP-1 can serve as an immune-related biomarker for the diagnosis and disease severity of trauma and sepsis. It may activate the IL6 JAK-STAT3 signaling pathway and promote CD8 T-cell depletion to trigger traumatic sepsis.

## Introduction

Trauma is a major global health concern that leads to the death of approximately 5 million people worldwide each year. It is the leading cause of death among people aged <45 years. Moreover, it has the highest real economic and social cost of any disease (1, 2). Trauma initiates a complex immune response in the minutes after the initial insult, and patients subjected to severe trauma are at risk of developing sepsis (3). The lack of timely diagnosis and treatment of the pathological changes and the rapidly deteriorating health condition, organ dysfunction, and microcirculatory hypoperfusion and hypotension after trauma can often lead to the development of septic shock (4). Despite the use of novel antibiotics and resuscitation therapies, trauma and sepsis remain the most lethal diseases, with sepsis responsible for one-third of in-hospital deaths (5). However, early prediction of the onset and progression of traumatic sepsis as well as early intervention for high-risk patients can effectively reduce its incidence and mortality. Considering that trauma and sepsis are highly complex conditions, with often challenging clinical assessments, the use of biomarkers, especially immune-related biomarkers, can be a viable method for the rapid diagnosis and the identification of high-risk patients.

The complement system is triggered by apoptosis or injury and acts as a bridge between the innate and adaptive immune systems. It plays a critical role in defense against microbial infections by mediating opsonization, sequestration, and lysis of pathogens (6, 7). However, complement activation is unregulated in sepsis, trauma, and sepsis-related coagulopathies (8). Complement activation can be achieved by three distinct pathways, namely, the alternative, classical, and lectin pathways, among which the lectin pathway is the most researched (9). Larsen et al. (10) found that mannose-binding lectin (MBL)-associated serine protease-1 (MASP-1), an important component of the lectin pathway, is associated with impaired coagulation in septic shock patients and that it can serve as a promising candidate biomarker for sepsis-induced disseminated intravascular coagulation. Increased MASP-1 activation and consumption are associated with severe coagulation disturbances in sepsis. Blasio et al. (11) found that MBL, ficolin-2, ficolin-3, MASP-2, and MASP-3 were significantly increased in traumatic brain injury (TBI) contusions compared to non-TBI tissues, suggesting that the lectin pathway is involved in the post-traumatic pathophysiology of human TBI. However, the role of MASP-1 in trauma and post-traumatic sepsis remains largely elusive. Compared to the other complement components, MASP-1 has multifaceted substrates for complement and non-complement proteins. In addition to activating MASP-2 and MASP-3, MASP-1 is involved in bradykinin release, coagulation activation, and endothelium–platelet endothelium–neutrophil interactions, thus mediating proinflammatory and procoagulant reactions (12). Therefore, determining the role of MASP-1 in the development of trauma and sepsis is of particular clinical relevance as it can facilitate their early detection and treatment.

The diagnosis of post-traumatic sepsis is notoriously difficult as trauma and sepsis have a variety of etiologies and trauma patients are in a state of “sterile inflammation” (13). Interestingly, similar genomic alterations have been observed in patients, following a range of pathophysiological insults, including trauma and sepsis (14). The availability of a large number of transcriptome databases provides an unprecedented opportunity to address these knowledge gaps. We hypothesized that MASP-1 levels are associated with the initiation and severity of trauma and sepsis. Therefore, in the present study, we used transcriptome analysis of the Gene Expression Omnibus (GEO) datasets and *in vitro* experiments to comprehensively investigate the expression pattern, biological functions, and diagnostic value of MASP-1 as well as the association between MASP-1 expression and clinicopathologic characteristics in trauma and sepsis. Thereafter, we systematically analyzed the association between MASP-1, immune cell infiltration (ICI), and immune-related molecular pathways, and based on these results, we proposed an underlying mechanism of the regulatory role of MASP-1 in the occurrence of trauma and sepsis.

## Materials and methods

### Dataset collection and processing

We obtained seven RNA-sequencing (RNA-seq) datasets, consisting of five sepsis datasets (GSE54514, *n* = 163; GSE57065, *n* = 107; GSE95233, *n* = 124; GSE131761 *n* = 129; and GSE154918, *n* = 93), two trauma datasets (GSE11375, *n* = 184 and GSE64711 *n* = 495), and one trauma and sepsis dataset (GSE69063, *n* = 130), and the corresponding clinical information from the GEO database.^1^ Datasets that met the following criteria were included in our study: (1) organism: *Homo sapiens*; (2) critical illnesses: sepsis or trauma; (3) sample size ≥90; (4) age: ≥ 18 years old; (5) expression profiling method: array or high throughput sequencing; (6): injury severity score (ISS) (based on site of trauma, types of trauma, circulation, consciousness, and breathing): > 9 (for trauma RNA-seq dataset); and (7) sepsis diagnosis criteria: according to the Second/Third International Consensus Definitions for Sepsis and Septic Shock (for sepsis RNA-seq dataset) (15). Detailed information on these cohorts is summarized in Table 1. Notably, the GSE69063 dataset exclusively included patients with head trauma (generally speaking, head trauma accounts for approximately 10–20% of all injuries, is prone to sepsis, and is the leading cause of trauma-related deaths). Gene expression matrices from each cohort were individually log-2 transformed, background corrected, robust multiarray averaging normalized using the R package “affy,” and the batch effect was removed using the Bayesian method ComBat in the R package “sva” (16).

**Table 1:** Sepsis and trauma datasets included in the study.

Accession	Cohort description	Timing of gene expression profiling	Country	Normal/Control sample	Sepsis/Trauma sample	Dataset website
GSE54514	Sepsis	Day1/2/3/4/5 of ICU admission	Australia	36	127	https://www.ncbi.nlm.nih.gov/geo/query/acc.cgi?acc=GSE54514
GSE57065	Septic shock	0 h/24 h/48 h of ICU admission	France	25	82	https://www.ncbi.nlm.nih.gov/geo/query/acc.cgi?acc=GSE57065
GSE95233	Septic shock	Day1/3 of ICU admission	France	22	102	https://www.ncbi.nlm.nih.gov/geo/query/acc.cgi?acc=GSE95233
GSE131761	Septic shock	On ICU admission	Spain	15	114	https://www.ncbi.nlm.nih.gov/geo/query/acc.cgi?acc=GSE131761
GSE154918	Septic shock	Day 1 of ICU admission	Germany	40	53	https://www.ncbi.nlm.nih.gov/geo/query/acc.cgi?acc=GSE154918
GSE11375	Trauma	Within 12 h of hospital admission	USA	26	158	https://www.ncbi.nlm.nih.gov/geo/query/acc.cgi?acc=GSE11375
GSE64711	Severely injured patients with hemorrhagic shock	Within 12 h of injury	USA	17	478	https://www.ncbi.nlm.nih.gov/geo/query/acc.cgi?acc=GSE64711
GSE69063	Sepsis + Head trauma	0 h/1 h/3 h of ICU admission	Australia	33	57/30	https://www.ncbi.nlm.nih.gov/geo/query/acc.cgi?acc=GSE69063

ICU, intensive care medicine; USA, United States of America.

### Clinical specimen selection, collection, and pre-processing

A total of 63 adult patients, including 37 traumatic sepsis patients and 26 control patients (postoperative patients), were recruited at the First Affiliated Hospital of Hainan Medical University (Hainan, China) from October 2020 to September 2023. Traumatic sepsis patients were trauma patients who developed sepsis within a few days of the injury. The adult trauma patients with injury severity score (ISS) (based on site of trauma, types of trauma, circulation, consciousness, and breathing) higher than 9 were recruited in the study (2). Sepsis or septic shock was diagnosed according to the Third International Consensus Definitions for Sepsis and Septic Shock (15). Patients with traumatic sepsis who died within 48 h of admission were excluded. The clinicopathologic baseline characteristics, including age, procalcitonin (PCT) level, C-reaction protein (CRP) level, sequential organ failure assessment (SOFA) score, acute physiology and chronic health evaluation (APACHE)-II score, and routine blood report of each patient were extracted from the hospital information system. All the procedures involving human participants were approved by the Institutional Review Board (Ethics Committee) of the First Affiliated Hospital of Hainan Medical University (Approval No. 2022-L-46). Peripheral blood samples were taken into two 4 mL ethylenediaminetetraacetic acid (EDTA)-treated tubes (BD, United Kingdom) from each patient at the time of admission. One blood sample was processed within 4 h to obtain peripheral blood mononuclear cells (PBMCs), while the other was kept on ice and processed within 40 min to obtain plasma by centrifugation (3,000 *g* for 8 min). Both the samples were stored at −80°C for real-time quantitative polymerase chain reaction (RT-qPCR) and enzyme-linked immunosorbent assay (ELISA), respectively.

### *MASP-1* expression analysis

*MASP-1* expression levels were comprehensively analyzed between the trauma/sepsis and control samples in the multi-transcriptome data. Thereafter, principal component analysis (PCA) was performed to assess the discrimination ability of *MASP-1* expression (sepsis vs. control and trauma vs. control) in multiple datasets.

### Analysis of the diagnostic value of *MASP-1*

We assessed the diagnostic performance of the *MASP-1* in multiple gene expression profiles and at multiple time points. Additionally, we compared the *MASP-1* expression levels in different trauma/sepsis subgroups and investigated its correlation with clinical features, such as APACHE-II score and neutrophil levels.

### Analysis of the biological functions of MASP-1 in trauma and sepsis

To minimize sample heterogeneity and increase homogeneity, we selected the GSE69063 cohort, consisting of 33 control, 57 sepsis, and 30 head trauma patients, to explore the shared biological functions of MASP-1 in trauma and sepsis. Thereafter, differential gene expression analysis was conducted between trauma/sepsis and control samples using the R package “limma,” at an adjusted *p* < 0.05 (Benjamini–Hochberg method) and |log2 fold-change (FC)| >1 for identifying differentially expressed genes (DEGs), and Venn diagrams were used to visualize the shared DEGs. Thereafter, Pearson’s correlation analysis was conducted between *MASP-1* and shared DEGs in trauma and sepsis at *p* < 0.05, and Venn diagrams were used to visualize the shared MASP-1-related DEGs. Finally, the shared MASP-1-related DEGs were subject to gene ontology (GO) and Kyoto Encyclopedia of Genes and Genomes (KEGG) enrichment analysis using the R package “clusterProfiler,” and the results were visualized using the R package “dotplot.”

### RNA isolation and RT-qPCR

Total RNA was extracted from the PBMCs using the Illustra™ RNASpin RNA Isolation Kit (GE Healthcare, United States), according to the manufacturer’s protocol. Thereafter, the total RNA was reverse transcribed using the Tetro cDNA Synthesis Kit (Bioline, United Kingdom). qRT-PCR was performed on a CFX96 Touch Real-Time PCR detection system (Bio-Rad, United States). The primers were designed using the NCBI Primer-BLAST primer design tool and synthesized by IDT (Coralville, United States) (Supplementary material 1).

### ELISA

MASP-1 concentration in plasma samples was detected using a MASP-1 ELISA kit (R&D Company, United States), according to the manufacturer’s instructions.

### Analysis of ICI in trauma and sepsis

The immune cell fractions in the GSE69063 cohort were estimated by the CIBERSORTx tool^2^ and single-sample gene set enrichment analysis (ssGSEA) (17). Thereafter, we conducted a comparative analysis of the immune cell fractions between the trauma/sepsis and control samples. Finally, Spearman’s correlation analysis was performed to evaluate the association between MASP-1 expression and immune cells in trauma and sepsis.

### Analysis of immune and molecular pathways in trauma and sepsis

Gene set variation analysis (GSVA) was conducted to determine the enrichment degree of specific biological processes, such as cell death-related pathways, immune-related pathways, and inflammation-related pathways, in trauma and sepsis (18) (Supplementary material 2). Subsequently, we investigated the differences in the biological processes between the trauma/sepsis and control samples. Finally, we performed a correlation analysis to determine the association between MASP-1 expression and biological pathways in trauma and sepsis.

### Correlation analysis between MASP-1, immune cells, and biological pathways in trauma and sepsis

We conducted further analysis to illustrate the underlying mechanism of MASP-1 in the occurrence of trauma and sepsis. First, single-cell data and transcriptional data from the HPA database^3^ were used to analyze MASP-1 expression in immune cells (19). Thereafter, Spearman’s correlation analysis was performed to explore the correlation between immune cells and common biological pathways. Finally, the Mantel test was used to investigate the correlation between MASP-1, specific immune cells, and biological pathways in trauma and sepsis.

### Statistical analysis

Statistical analysis was performed using the R software (v4.0.4). The Wilcoxon test and Kruskal–Wallis test were used to analyze the statistical differences between the two groups. A receiver operating characteristic (ROC) curve with an area under the curve (AUC) was used to evaluate the diagnostic ability of MASP-1 in trauma and sepsis. The AUCs of MASP-1, PCT, and CRP were compared using the R package “pROC.” The *p* < 0.05 was considered statistically significant unless specified otherwise.

## Results

### *MASP-1* expression analysis

**Figure 1:**
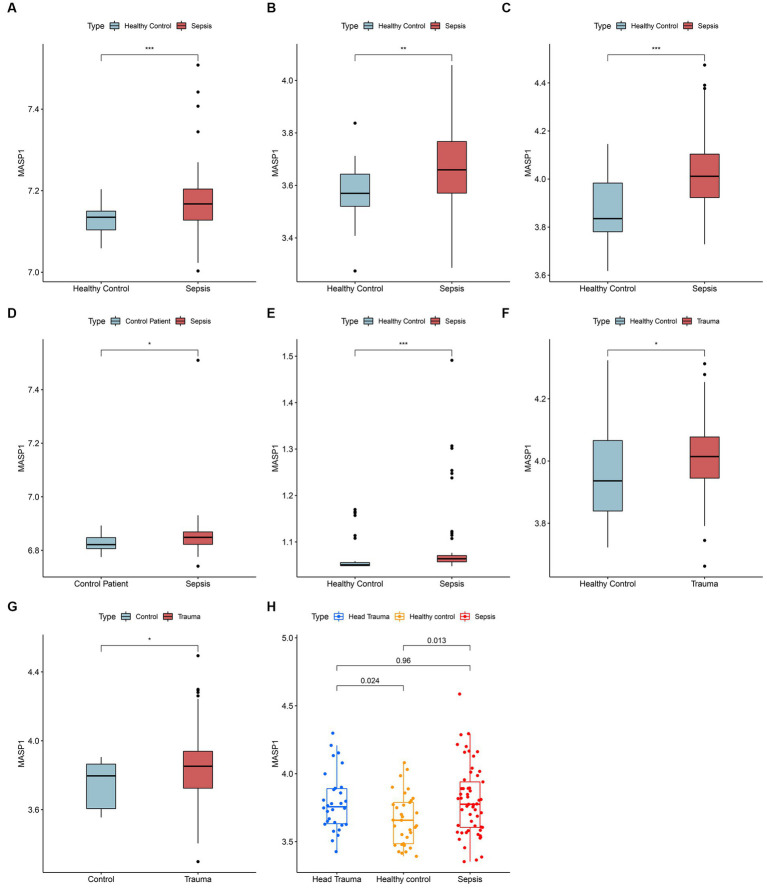
Landscape of expression variation of MASP-1 in sepsis and trauma. **(A–E)**: Differential expression of MASP-1 between sepsis samples and healthy control/control patient samples in five GEO datasets. **(A)**: GSE54514 datasets; **(B)**: GSE57065 datasets; **(C)**: GSE95233 datasets; **(D)**: GSE131761 datasets; **(E)**: GSE154918 datasets. **(F,G)**: Differential expression of MASP-1 between trauma samples and healthy control/control patient samples in five GEO datasets. **(F)**: GSE11375 datasets; **(G)**: GSE64711 datasets. The asterisks indicate a significant statistical *p*-value calculated using the Wilcoxon test (*p < 0.05; **p < 0.01; ***p < 0.001). **(H)**: Differential expression of MASP-1 between trauma samples, sepsis samples, and healthy control samples in GSE69063 datasets. The *p*-value was calculated using the Kruskal–Wallis test.

**Figure 2:**
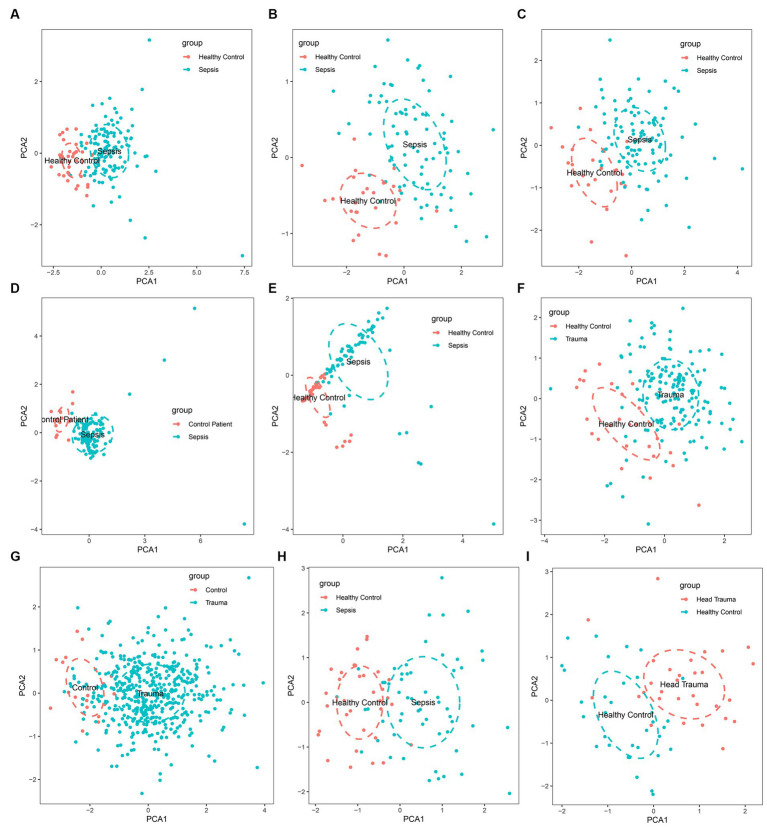
Principal component analysis for the expression of MASP-1 to distinguish sepsis/trauma patients from healthy control/control patients in multi-transcriptome cohorts. **(A)**: GSE54514 datasets; **(B)**: GSE57065 datasets; **(C)**: GSE95233 datasets; **(D)**: GSE131761 datasets; **(E)**: GSE154918 datasets; **(F)**: GSE11375 datasets; **(G)**: GSE64711 datasets; **(H)**: GSE69063 datasets; **(I)**: GSE69063 datasets.

The gene expression analysis of the trauma and sepsis cohorts from the GEO database revealed that the mRNA levels of *MASP-1* were significantly (*p* < 0.05) upregulated in sepsis (Figures 1A–E) and trauma (Figures 1F,G) samples compared to the control samples. Additionally, *MASP-1* expression was significantly higher in sepsis and head trauma samples compared with the healthy controls in the GSE69063 cohort (Figure 1H). Interestingly, PCA revealed that *MASP-1* expression could completely distinguish trauma/sepsis samples from the control patient/healthy samples (Figures 2A–I).

### The diagnostic value of MASP-1 and risk stratification

**Figure 3:**
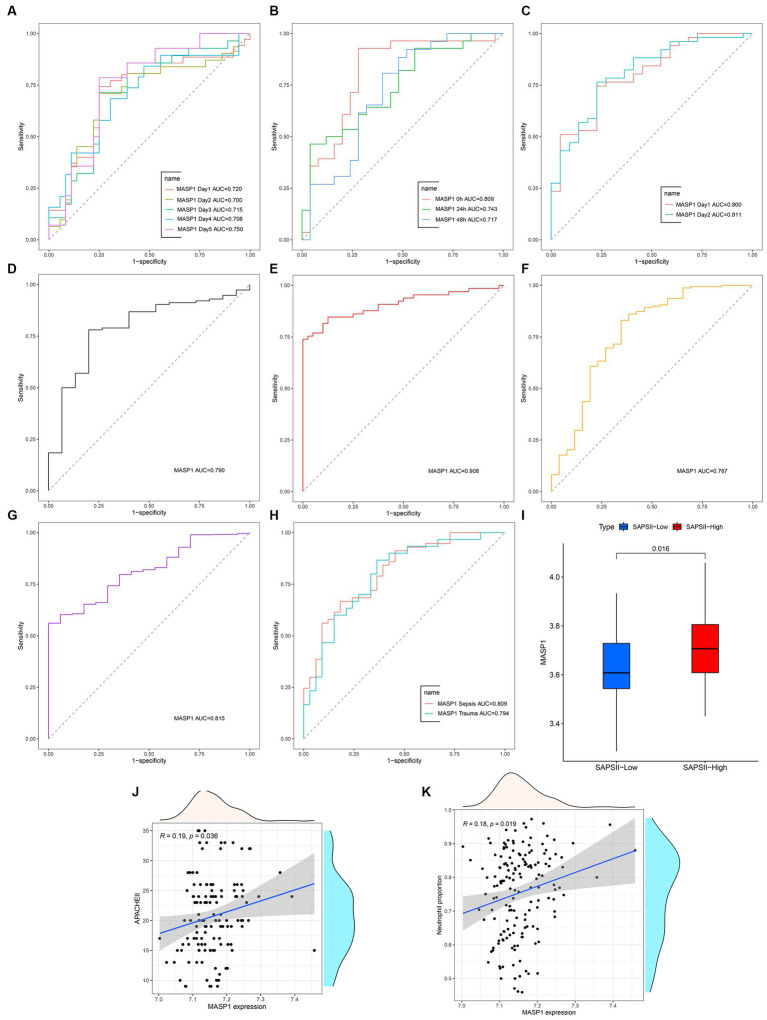
The clinical value of MASP-1 in multi-transcriptome datasets from sepsis and trauma. **(A–H)**: ROC curves analyzed the diagnostic accuracy of MASP-1 in multiple datasets and time points. **(A)**: GSE54514 datasets; **(B)**: GSE57065 datasets; **(C)**: GSE95233 datasets; **(D)**: GSE131761 datasets; **(E)**: GSE154918 datasets; **(F)**: GSE11375 datasets; **(G)**: GSE64711 datasets; **(H)**: GSE69063 datasets. **(I)**: Comparison of MASP-1 expression between SAPS II—low and SAPS II—high in GSE57065 datasets. The *p-*value was calculated using the Wilcoxon test **(J,K)**. Correlation between MASP-1 expression and APACHE-II **(J)**, neutrophil proportion **(K)** in GSE54514 datasets. APACHE-II: Acute Physiology and Chronic Health Evaluation II. The correlation coefficient and *p*-value were calculated by Spearman’s correlation analysis.

MASP-1 exhibits excellent diagnostic ability in multiple sepsis (AUC > 0.70) and trauma (AUC > 0.75) datasets (Figures 3A–H). In addition, MASP-1 showed favorable diagnostic values for sepsis at multiple time points (Figures 3A–C). In the GSE57065 dataset, *MASP-1* expression was significantly upregulated in the simplified acute physiology score (SAPS) II—high group compared to the SAPS II—low group (*p* = 0.016) (Figure 3I). Additionally, in the GSE54514 dataset, *MASP-1* expression was significantly positively associated with APACHE-II (*R* = 0.19, *p* = 0.036) (Figure 3J) and neutrophil levels (*R* = 0.19, *p* = 0.019) (Figure 3K).

### Biological functions of MASP-1 in trauma and sepsis

**Figure 4:**
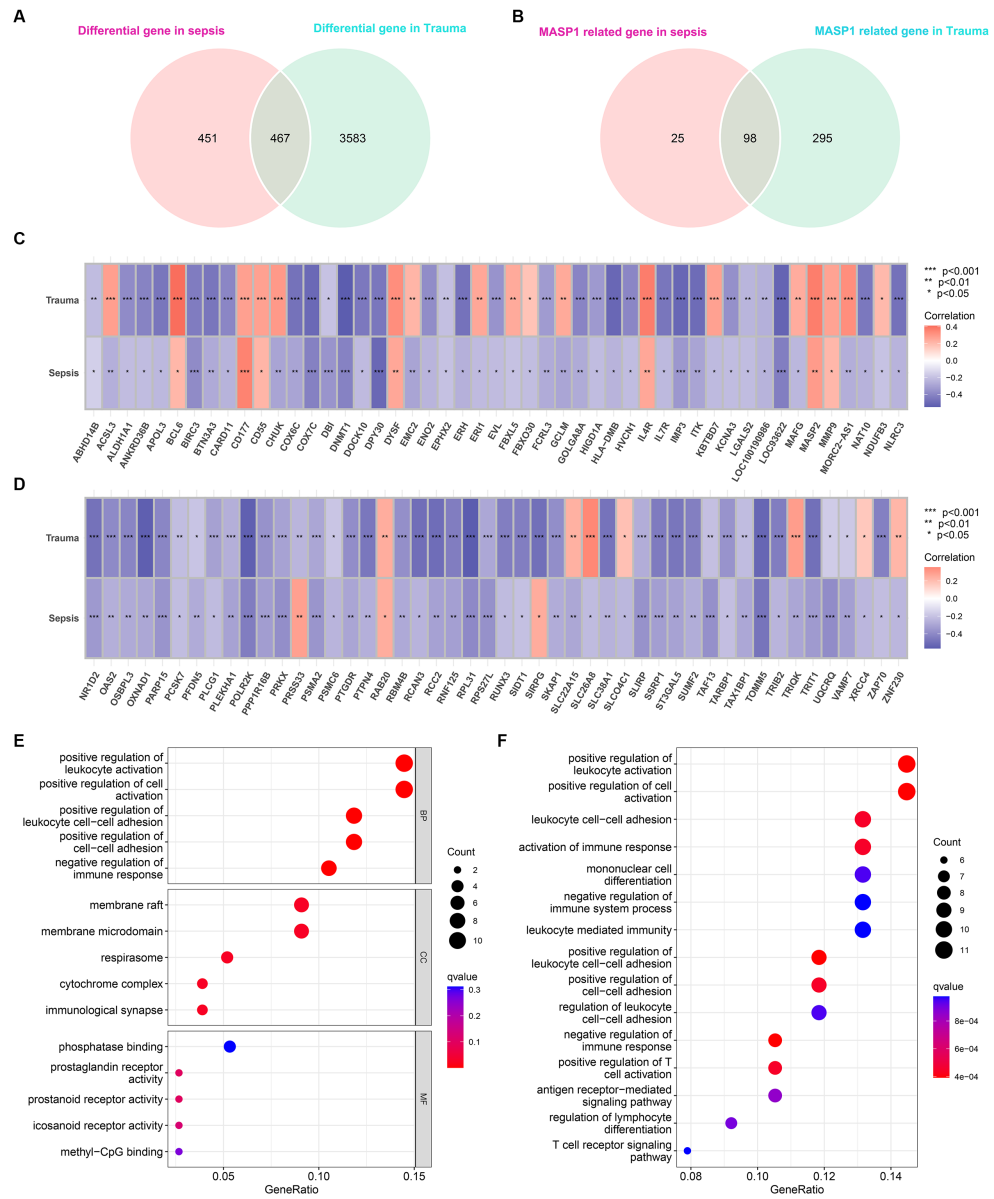
Identification of common MASP-1-related differentially expressed genes (DEGs) in sepsis and trauma as well as functional enrichment analysis. **(A)**: Venn diagrams present the shared DEGs in sepsis and trauma. **(B)**: Venn diagrams present the shared MASP-1-related DEGs in sepsis and trauma. **(C,D)**: Correlation between MASP-1 expression and shared DEGs. The correlation coefficient and *p*-value were calculated by Spearman’s correlation analysis. **(E,F)**: Functional enrichment analysis of MASP-1-related DEGs in sepsis and trauma. **(E)**: Gene ontology (GO) analysis on the biological process (BP), cellular component (CC), and molecular function (MF); **(F)**: Kyoto Encyclopedia of Genes and Genomes (KEGG) pathways.

Differential gene expression analysis identified 918 and 4,050 DEGs (*p* < 0.05 and |log2 FC (fold-change)| >1) in trauma and sepsis samples, respectively, among which 467 DEGs were common between the two (Figure 4A). Additionally, Pearson’s correlation analysis identified 123 and 393 MASP-1-related DEGs in sepsis and trauma samples, respectively, among which 98 were shared between the two (Figures 4B–D).

The GO enrichment analysis of the shared MASP-1-related DEGs revealed the top five significant clusters of enriched sets in biological process (BP), molecular function (MF), and cellular component (CC) categories (Figure 4E), such as positive regulation of leukocyte activation, positive regulation of cell activation, positive regulation of leukocyte cell–cell adhesion, positive regulation of cell–cell adhesion, negative regulation of immune response, membrane raft, phosphatase binding, and prostaglandin receptor activity. Additionally, the KEGG enrichment analysis of the shared MASP-1-related DEGs revealed that the top five enriched KEGG pathways were positive regulation of leukocyte activation, positive regulation of cell activation, leukocyte cell–cell adhesion, activation of the immune response, and mononuclear cell differentiation (Figure 4F).

### Preliminary experimental validation

**Figure 5:**
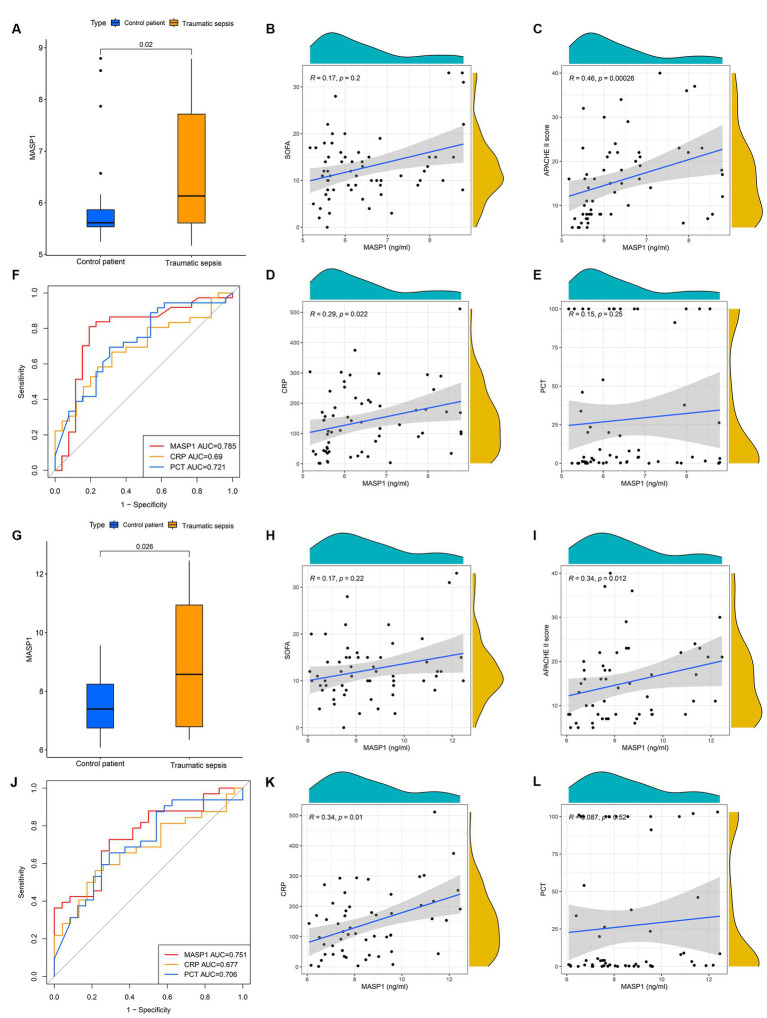
The clinical application of MASP-1 in traumatic sepsis was validated by real-time quantitative polymerase chain reaction (RT-qPCR) and enzyme-linked immunosorbent assay (ELISA). **(A–F)**: The results of RT-qPCR. **(A)**: Comparison of mRNA levels of MASP-1 between traumatic sepsis and control patients. The *p-*value was calculated using the Wilcoxon test. **(B–E)**: correlation between MASP-1 expression and SOFA **(B)**, APACHE-II **(C)**, CRP **(D)**, and PCT **(E)**. The correlation coefficient and *p*-value were calculated by Spearman’s correlation analysis. **(F)**: ROC curves compared the diagnostic efficacy of MASP-1 expression, PCT, and CRP. **(G–L)**: The results of ELISA. **(G)**: Comparison of MASP-1 proteins between traumatic sepsis and control patients. The *p-*value was calculated using the Wilcoxon test. **(H–K)**: Correlation between MASP-1 proteins and SOFA **(H)**, APACHE-II **(I)**, CRP **(J)**, and PCT **(K)**. The correlation coefficient and *p*-value were calculated by Spearman’s correlation analysis. **(L)**: ROC curves compared the diagnostic efficacy of MASP-1 proteins, PCT, and CRP.

To further verify the MASP-1 expression levels in trauma and sepsis, we performed qRT-PCR and ELISA of 63 clinical blood specimens. The qRT-PCR analysis revealed that *MASP-1* expression was significantly upregulated in the traumatic sepsis patients compared to the control patients (*p* = 0.02) (Figure 5A). Similarly, ELISA showed that MASP-1 was significantly higher in the traumatic sepsis patients compared with the control patients (*p* = 0.026) (Figure 5G). Moreover, both mRNA and protein expression levels of MASP-1 were significantly positively correlated with APACHE-II (*R* = 0.46, *p* < 0.001 and *R* = 0.34, *p* = 0.012) (Figures 5C,I) and CRP (*R* = 0.29, *p* = 0.022 and *R* = 0.34, *p* = 0.01) (Figures 5D,K) but not correlated with SOFA (*R* = 0.17, *p* = 0.2 and *R* = 0.17, *p* = 0.22) (Figures 5B,H) and PCT (*R* = 0.15, *p* = 0.25 and *R* = 0.087, *p* = 0.52) (Figures 5E,L). In addition, MASP-1 mRNA and protein expression (AUC = 0.785 and 0.751) showed a superior diagnostic ability compared to both PCT (AUC = 0.721, *p* = 0.045 and AUC = 0.706; *p* = 0.048) and CRP (AUC = 0.690, *p* = 0.015 and AUC = 0.677; *p* = 0.02) (Figures 5F,J).

### ICI in trauma and sepsis

**Figure 6:**
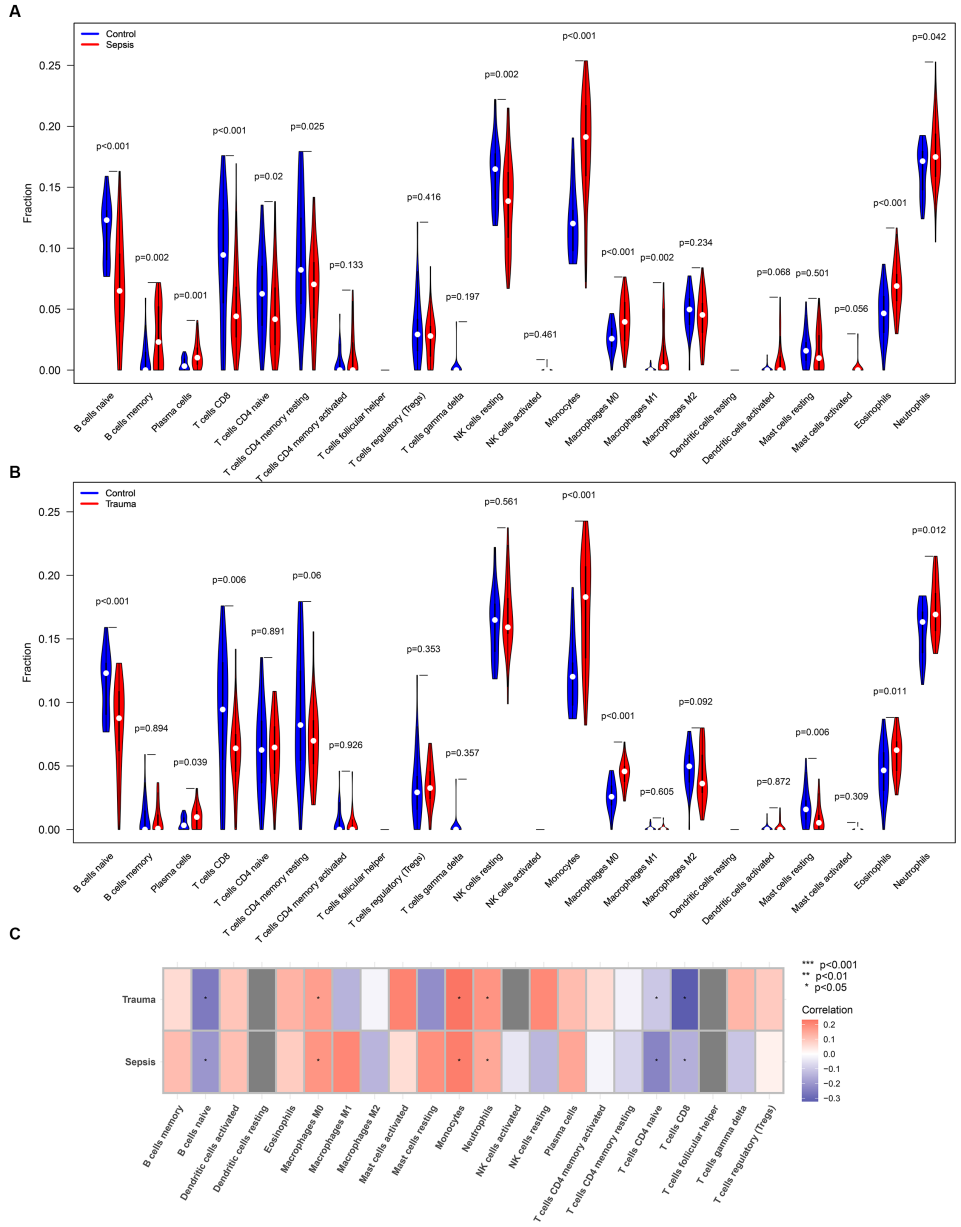
Analyzing the correlation between MASP-1 and common infiltrating immune cells in sepsis and trauma datasets based on the CIBERSORTx tool. **(A)** Comparison of infiltrating immune cells between sepsis samples and healthy control samples. The *p-*value was calculated using the Wilcoxon test. **(B)** Comparison of infiltrating immune cells between trauma samples and healthy control samples. The *p-*value was calculated using the Wilcoxon test. **(C)** Correlation between MASP-1 and immune cells sepsis and trauma. The correlation coefficient and *p*-value were calculated by Spearman’s correlation analysis.

We used the CIBERSORTx tool and ssGSEA algorithm to determine the relative abundance of infiltrating immune cells in the immune microenvironment of trauma and sepsis patients to determine the shared immune cells. The CIBERSORTx results revealed that memory B cells (*p* = 0.002), plasma cells (*p* = 0.001), monocytes (*p* < 0.001), M0 macrophages (*p* < 0.001), M1 macrophages (*p* = 0.002), eosinophils (*p* < 0.001), and neutrophils (*p* = 0.042) were more abundant, while naive B cells (*p* < 0.001), CD8 T cells (*p* < 0.001), naive CD4 T cells (*p* = 0.02), memory resting CD4 T cells (*p* = 0.025), and resting NK cells (*p* = 0.002) were significantly lower in sepsis samples compared with the control samples (Figure 6A). Additionally, plasma cells (*p* = 0.039), monocytes (*p* < 0.001), M0 macrophages (*p* < 0.001), eosinophils (*p* = 0.011), and neutrophils (*p* = 0.012) were relatively higher, while naive B cells (*p* < 0.001), CD8 T cells (*p* = 0.006), and resting mast cells (*p* = 0.006) were relatively lower in trauma samples compared with the control samples (Figure 6B). Furthermore, Spearman’s correlation analysis revealed that MASP-1 expression was significantly negatively correlated with naive B cells, naive CD4 T cells, and CD8 T cells and significantly positively correlated with M0 macrophages, monocytes, and neutrophils in trauma and sepsis (Figure 6C).

**Figure 7:**
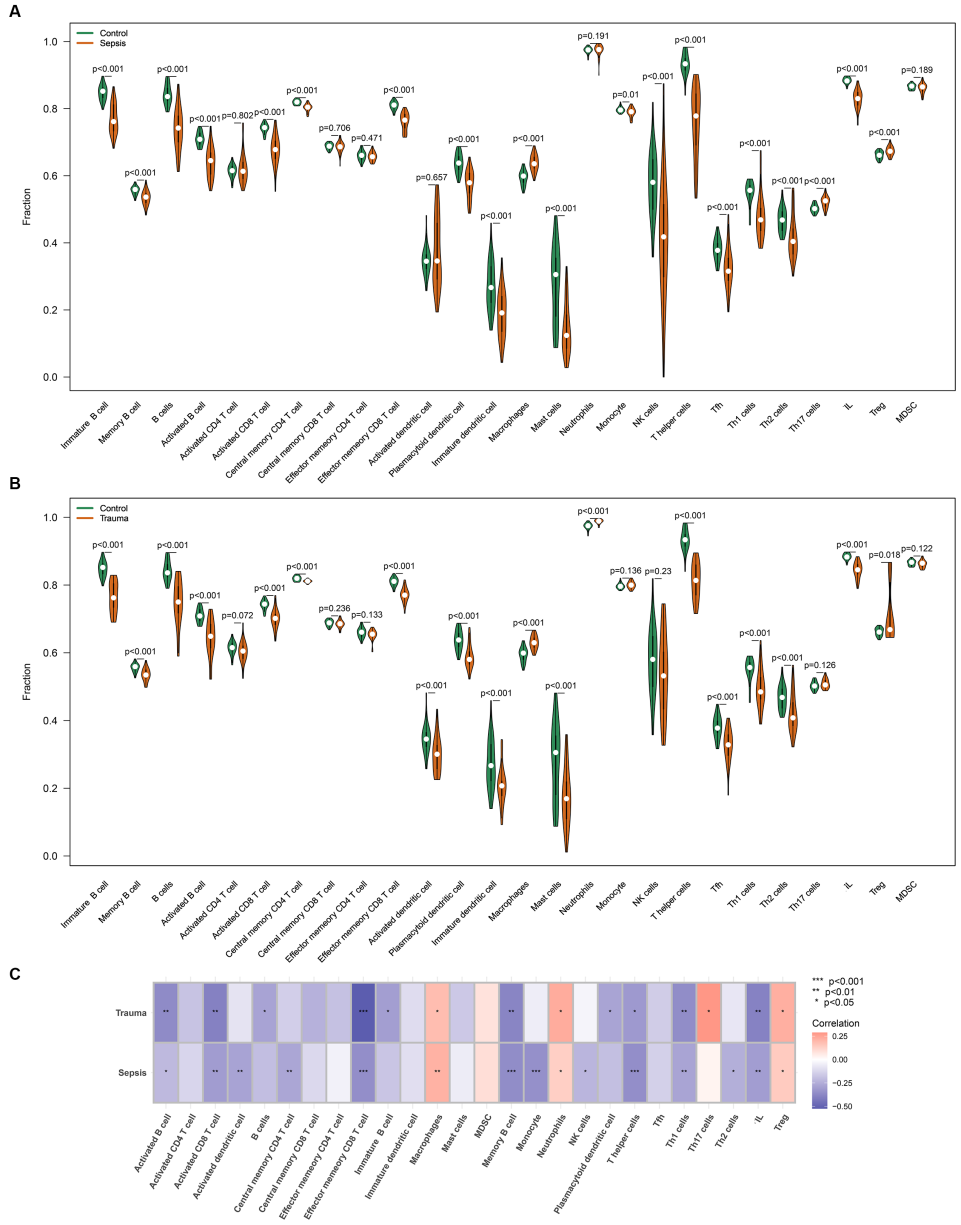
Analyzing the correlation between MASP-1 and common infiltrating immune cells in sepsis and trauma datasets based on ssGSEA algorithms. **(A)** Comparison of infiltrating immune cells between sepsis samples and healthy control samples. The *p-*value was calculated using the Wilcoxon test. **(B)** Comparison of infiltrating immune cells between trauma samples and healthy control samples. The *p-*value was calculated using the Wilcoxon test. **(C)** Correlation between MASP-1 and immune cell sepsis and trauma. The correlation coefficient and *p*-value were calculated by Spearman’s correlation analysis.

Similarly, the ssGSEA revealed that infiltrating lymphocytes (ILs) (*p* < 0.05), dendritic cells (*p* < 0.05), CD8 T cells (*p* < 0.05), CD4 T cells (*p* < 0.05), and B cells (*p* < 0.05) were relatively higher, while macrophages (*p* < 0.05) and T regulatory cells (Tregs) (*p* < 0.05) were significantly lower in trauma/sepsis samples compared to the control samples (Figures 7A,B). Further correlation analysis revealed that MASP-1 expression was significantly negatively associated with B cells, CD8 T cells, ILs, T helper (Th) cells, and Th1 cells and significantly positively correlated with macrophages, neutrophils, and Tregs in trauma and sepsis (Figure 7C).

### ICI in traumatic sepsis patients based on routine blood report

We analyzed immune cell levels in the blood samples of traumatic sepsis patients. The results revealed that the percentage of neutrophils (*p* < 0.05), monocytes (*p* < 0.05), and lymphocytes (*p* < 0.05) were significantly higher in traumatic sepsis patients than in the control patients (Supplementary Figure S1), consistent with the results of CIBERSORTx and ssGSEA.

### Immune and molecular pathways in trauma and sepsis

**Figure 8:**
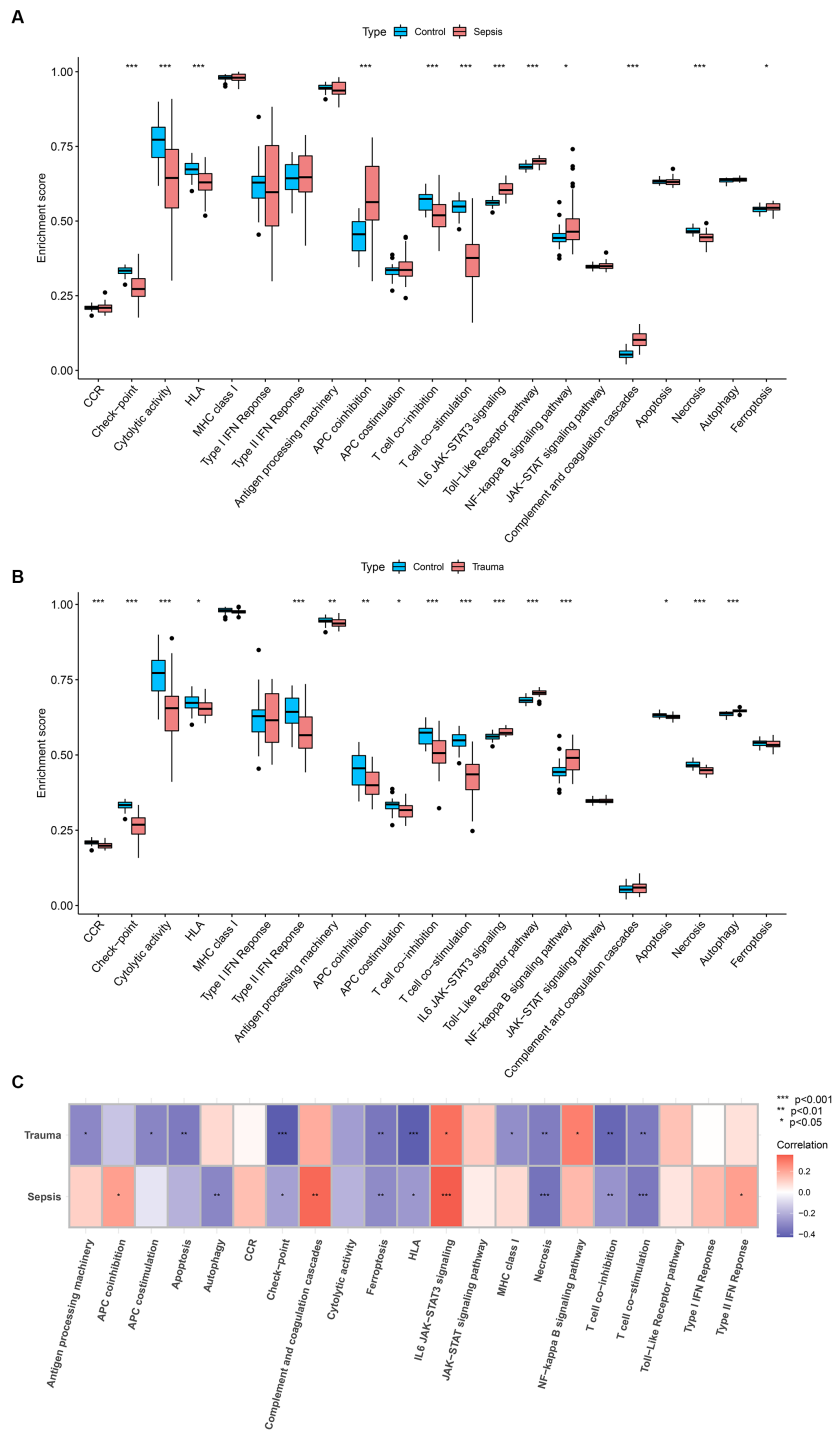
Analyzing the correlation between MASP-1 and common biological pathways in sepsis and trauma based on Gene set variation analysis (GSVA). **(A)** Comparison of biological pathways between sepsis samples and healthy control samples. The *p-*value was calculated using the Wilcoxon test. **(B)** Comparison of biological pathways between trauma samples and healthy control samples. The *p-*value was calculated using the Wilcoxon test. **(C)** Correlation between MASP-1 and biological pathways sepsis and trauma. The correlation coefficient and *p*-value were calculated by Spearman’s correlation analysis.

GSVA was used to determine the enrichment score of specific biological processes in trauma and sepsis to determine the shared molecular pathways that may be involved in their pathophysiology. The results revealed that APC co-inhibition, interleukin-6/Janus kinase/signal transducer and activator of transcription 3 (IL6/JAK/STAT3) signaling, Toll-like receptor (TLR) pathway, nuclear factor–kappa B (NF-κB) signaling pathway, complement and coagulation cascades, and ferroptosis were enriched, while checkpoint, cytolytic activity, HLA, T-cell co-inhibition, T-cell co-stimulation, and necrosis were inhibited in the sepsis samples compared with the control samples (Figure 8A). Additionally, the results revealed that IL6/JAK/STAT3 signaling, TLR pathway, NF-κB signaling pathway, and autophagy were enriched, while CCR, checkpoint, cytolytic activity, HLA, type II interferon response, antigen processing machinery, APC co-inhibition, APC co-stimulation, T-cell co-inhibition, T-cell co-stimulation, apoptosis, and necrosis were suppressed in the trauma samples compared with the control samples (Figure 8B). Further correlation analysis revealed that MASP-1 expression was significantly positively associated with IL6/JAK/STAT3 signaling and significantly negatively correlated with checkpoint, ferroptosis, HLA, T-cell co-inhibition, T-cell co-stimulation, and necrosis in trauma and sepsis (Figure 8C).

### Correlation between MASP-1, immune cells, and biological pathways in trauma and sepsis

**Figure 9:**
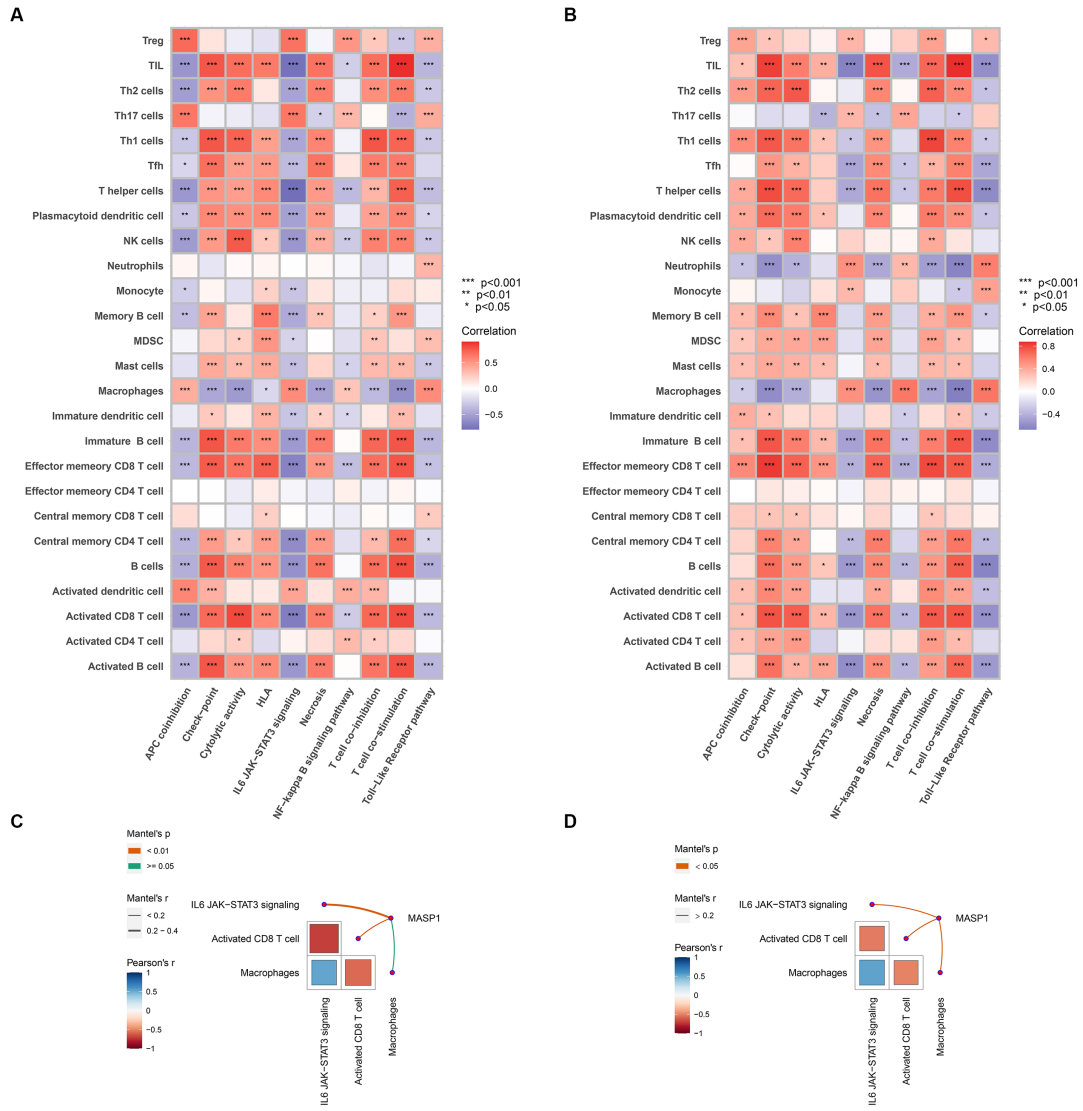
Analyzing the correlation between MASP-1, immune cells, and biological pathways in sepsis and trauma. **(A,B)**: Correlation between immune cells and shared biological pathways sepsis **(A)** and trauma **(B)**. The correlation coefficient and *p*-value were calculated by Spearman’s correlation analysis. **(C,D)**: Correlation between MASP-1, immune cells, and biological pathways in sepsis **(C)** and trauma **(D)**. The correlation coefficient and *p*-value were calculated by the Mantel test.

To illustrate the potential mechanism of MASP-1 in the onset of trauma and sepsis, we performed Spearman’s correlation analysis between immune cells and common biological pathways. We found that shared biological pathways, especially checkpoint, cytolytic activity, IL6/JAK/STAT3 signaling, and TLR pathway were significantly correlated with most of the infiltrating immune cells in sepsis (Figure 9A) and trauma (Figure 9B). Monaco and Schmiedel datasets from the HPA showed that MASP-1 was primarily expressed in T cells (Supplementary Figure S2). The results of the Mantel test revealed that MASP-1 expression was significantly negatively associated with the proportion of CD8 T cells (*p* < 0.05) and IL6/JAK/STAT3 signaling scores (*p* < 0.05), while IL6/JAK/STAT3 signaling score was significantly negatively correlated with the proportion of CD8 T cells (*p* < 0.05) in sepsis (Figure 9C) and trauma (Figure 9D).

## Discussion

In the present study, we comprehensively analyzed the role of MASP-1 in trauma and sepsis using five sepsis (GSE54514, GSE57065, GSE95233, GSE131761, and GSE154918), two trauma (GSE11375 and GSE64711), and one sepsis and trauma (GSE69063) GEO datasets. An in-depth analysis of these transcriptome datasets revealed that *MASP-1* was significantly upregulated in trauma/sepsis samples compared to the control samples. Moreover, MASP-1 exhibited excellent diagnostic ability (AUC > 0.7) in multiple datasets and time points and could effectively distinguish trauma/sepsis samples from the control samples. Additionally, *MASP-1* expression was significantly positively correlated with the severity of trauma and sepsis (APACHE-II and neutrophil proportion). RT–qPCR and ELISA of clinical specimens further verified that MASP-1 expression was significantly higher in traumatic sepsis patients compared with the control patients. Further analysis revealed that MASP-1 expression was significantly positively associated with APACHE-II and CRP and that it showed superior diagnostic ability than CRP and PCT. The GO and KEGG enrichment analyses revealed that MASP-1 primarily mediated trauma/sepsis via the immune-related signaling pathway. Additionally, Spearman’s correlation analysis revealed that MASP-1 was significantly correlated with ICI and immune and molecular pathways. Transcriptional data and single-cell data from the HPA revealed that MASP-1 was predominantly expressed in T cells. Further correlation analysis revealed a close association between MASP-1 expression, the proportion of CD8 T cells, and IL6/JAK/STAT3 signaling scores in trauma and sepsis.

Trauma and sepsis are characterized by dysregulated responses to systemic and acute immune-inflammatory reactions, leading to exacerbated production of several inflammatory and anti-inflammatory mediators, such as cytokines, complements, and the coagulation cascade, responsible for organ dysfunction and coagulation disorders (20). Consequently, numerous potential biomarkers are generated by the acute dysregulation of multiple biochemical and cellular pathways in trauma and sepsis pathobiology. MASP-1 can activate a large number of complement and non-complement proteins, such as prothrombin and coagulation factors, and plays a critical role in regulating proinflammatory and procoagulant reactions (21). However, the effects of MASP-1 on post-traumatic sepsis remain unclear. We speculated that MASP-1 can identify early stages of post-traumatic sepsis and reflect the natural history of the traumatic sepsis condition. To our knowledge, this is the first study to apply transcriptomic profiling databases and *in vitro* experiments in all-cause trauma and sepsis to investigate the expression pattern, diagnostic value, and biological functions of MASP-1 and to explore the relationship between MASP-1 expression and clinicopathologic characteristics of trauma and sepsis.

Currently, several biomarkers, such as CRP (22), PCT (23), interleukin-28 (24), HLA-A Locus (25), vanin-1 (26), C5a (27), thrombin activatable fibrinolysis inhibitor (27), and microvesicles (MV) (28), are being used as diagnostic, predictive, and prognostic markers for post-traumatic sepsis. However, these biomarkers have some limitations. First, the diagnostic and prognostic performance of several makers, including CRP and PCT, is suboptimal for traumatic sepsis (22, 23). Second, previous studies primarily focused on single-center cohort studies or single ethnic groups that cannot be extended to the general population (24, 27); however, prospective multicenter cohort studies with multi-ethnic populations can improve biomarker generalizability. Third, the sample size in the previous studies was relatively small, which can reduce statistical power and influence the reliability of the results (26). Fourth, some markers require sophisticated techniques for detection, such as next-generation sequencing for HLA genotyping and flow cytometry for phenotypic analysis of MV, which can be challenging for clinical application. In this study, we analyzed multiple transcriptome datasets from different countries to perform a “systems-level analysis” of MASP-1 to derive meaningful and unbiased results and to identify homogenous patient subgroups deciphering disease heterogeneity and provide insights into shared biological processes and underlying mechanisms of trauma and sepsis under a variety of pathophysiological insults.

Differential gene expression analysis of the multi-transcriptome data revealed that the mRNA levels of *MASP-1* were significantly upregulated in trauma and sepsis samples compared to the control samples. A prospective cohort study by Larsen et al. (10), involving 36 septic shock patients, found that increased MASP-1 activation and consumption is associated with coagulation disturbances in septic shock patients. In this study, we found that MASP-1 showed superior diagnostic ability across multiple datasets and time points for trauma and sepsis and that it could effectively distinguish trauma/sepsis samples from control patient/healthy control samples. Furthermore, we observed a minor decrease in AUC values over time in various time point datasets, suggesting that MASP-1 may be suitable for the early identification of trauma and sepsis. This is possibly due to the initial activation and subsequent depletion of MASP-1 in trauma-induced sepsis. Moreover, qRT-PCR and ELISA results further confirmed that MASP-1 expression was significantly upregulated in traumatic sepsis patients than in control patients and that it exhibited superior diagnostic accuracy than general clinical biomarkers, such as CRP and PCT. Furthermore, correlation analysis revealed that MASP-1 was significantly positively associated with APACHE-II and CRP but not correlated with SOFA and PCT, consistent with a previous report by Larsen et al. (10), who also did not find any association between MASP-1/2 and SOFA score. Due to each mirroring different pathophysiological aspects, we propose the combination of MASP-1 expression and other clinical information (such as PCT) to further improve diagnostic efficiency and guide individualized patient management in the future. Altogether, our results indicate that MASP-1 can serve as a robust biomarker for early diagnosis of traumatic sepsis.

The GO and KEGG enrichment analyses of the shared MASP-1-related DEGs in trauma and sepsis revealed that MASP-1 was primarily involved in the positive regulation of leukocyte activation, positive regulation of cell activation, positive regulation of leukocyte cell–cell adhesion, positive regulation of cell–cell adhesion, activation of the immune response, and mononuclear cell differentiation, suggesting that MASP-1 plays an important role in immune regulation in trauma and sepsis. Previous studies found that MASP-1 participates in modulating cell adhesion and immune response. For instance, MASP-1 aids in the removal of microorganisms and/or tissue debris during injuries or infections by promoting the extravasation of soluble and cellular components of the immune system (29). Furthermore, MASP-1 enhances the adherence between neutrophil model cells (dPLB-985) and endothelial cells by altering the adhesion molecule pattern (upregulating E-selectin expression and downregulating ICAM-2) (30). Therefore, these results indicate that MASP-1 is involved in immune modulation in trauma and sepsis.

Based on these results, we investigated the ICI in trauma and sepsis samples. CIBERSORTx and ssGSEA results revealed that B cells, naive CD4 T cells, CD8 T cells, macrophages, monocytes, and neutrophils were abundant in both trauma and sepsis patients. Further correlation analysis revealed that MASP-1 expression was significantly associated with B cells, CD8 T cells, neutrophils, and macrophages in trauma and sepsis. Notably, *MASP-1* expression was significantly positively correlated with neutrophil proportion in the GSE54514 dataset. Consistent with our findings, Zador et al. (31) identified two consistent and distinct patient subgroups (increased neutrophil and suppressed neutrophil fraction groups) through the deconvolution of trauma and sepsis transcriptomics with significantly different clinical features. Abnormal CD8+ T-cell responses are major components of a dysregulated acquired immune response in trauma and sepsis. Previous research has shown that sepsis-associated apoptosis of CD8+ T cells leads to lymphopenia and immunosuppression as well as increased susceptibility to secondary infections, in late-stage sepsis patients (32). A recent study by Magatti et al. (33) found that TBI affected the differentiation ability of CD8 T cells in PBMCs, which are induced to differentiate into low cytotoxicity subtypes, such as memory-precursor effector CD8 T cells, leading to additional challenges, especially in the case of infections. Altogether, these results indicate that several immune cells, especially CD8 T cells, neutrophils, and macrophages, are involved in MASP-1-mediated trauma and sepsis.

Further investigation of the shared molecular pathways involved in the pathophysiology of MASP-1-induced trauma and sepsis revealed that IL6/JAK/STAT3 signaling, TLR pathway, NF-κB signaling pathway, checkpoint, HLA, T-cell co-inhibition, T-cell co-stimulation, and necrosis were shared between trauma and sepsis samples. Further correlation analysis revealed that MASP-1 expression was significantly correlated with IL6/JAK/STAT3 signaling, checkpoint, HLA, T-cell co-inhibition, T-cell co-stimulation, and necrosis. Notably, transcription data and single-cell data from the HPA found that MASP-1 was predominantly expressed in T cells. Additionally, the results of the Mantel test further verified the correlation between MASP-1 expression, CD8 T-cell proportion, and IL6/JAK/STAT3 signaling scores. Porro et al. (34) reported that disruption of the central nervous system homeostasis and microglia hyperactivation leads to an inflammatory storm following TBI. The JAK/STAT pathway can modulate the severity of this inflammatory storm to influence TBI prognosis. However, the effects of the JAK/STAT family on microglia are not fully understood. Chang et al. (35) showed that the JAK/STAT pathway is primarily involved in the pathogenesis of sepsis and that the inhibition of the JAK/STAT signaling pathway can improve the morphology of lung tissues in septic mice. Therefore, we propose that MASP-1 triggers traumatic sepsis by activating the IL6/JAK/STAT3 signaling pathways and promoting CD8 T-cell depletion after traumatic injury.

There are a few limitations in our study as mentioned below. First, our validation cohort was a single-center cohort with a limited sample size, which may not be generalizable; therefore, further validation using multicenter prospective cohorts with large sample sizes is necessary for the clinical application of MASP-1 in traumatic sepsis. Second, the general lack of corresponding prognostic data in transcriptomics limits the ability to better classify the patients to guide individualized therapy and further refine the prognostic value of MASP-1. Third, most of the results, such as ICI, biological processes, and underlying mechanisms, were yielded from independent trauma and sepsis transcriptome datasets, and although these results were from the same transcriptome datasets, they need to be further validated in the traumatic sepsis transcriptome dataset. Fourth, CIBERSORTx deconvolution and ssGSEA with metagenes may not accurately evaluate immune cell subpopulations, although the different methods and different datasets validate each other. Therefore, it is necessary to use single-cell RNA sequencing or fluorescence-activated cell sorting to verify our results in traumatic sepsis patients. Fifth, our study is limited to the transcriptome data in PBMCs; however, the use of other omics data (genomics, epigenetics, proteomics, and metabolomics) and mutual verification and supplementation can be implemented in further studies. Finally, further *in vitro* and *in vivo* experiments should be conducted in future studies to elucidate the regulatory mechanisms and functional roles of MASP-1, CD8 T cells, and IL6/JAK/STAT3 signaling pathways in trauma and sepsis.

## Conclusion

Our results indicate that MASP-1 is a potential immune-related biomarker for the diagnosis and disease severity of trauma and sepsis. It may activate the IL6/JAK/STAT3 signaling pathway and induce CD8 T-cell exhaustion to initiate trauma and sepsis.

## Data availability statement

The original contributions presented in the study are included in the article/Supplementary material, further inquiries can be directed to the corresponding author.

## Ethics statement

The studies involving human blood samples were reviewed and approved by the Research Ethics Committee of the First Affiliated Hospital of Hainan Medical University, and complied with the Declaration of Helsinki. The studies were conducted in accordance with the local legislation and institutional requirements. The participants provided their written informed consent to participate in this study.

## Author contributions

LX: Conceptualization, Funding acquisition, Investigation, Project administration, Resources, Software, Visualization, Writing – original draft, Writing – review & editing. SC: Data curation, Methodology, Writing – original draft, Writing – review & editing. WC: Investigation, Validation, Writing – review & editing. CZ: Formal analysis, Software, Writing – review & editing. ZH: Data curation, Methodology, Validation, Writing – review & editing. XD: Conceptualization, Data curation, Formal analysis, Investigation, Methodology, Software, Supervision, Validation, Visualization, Writing – original draft, Writing – review & editing.

## Glossary

**Table:** 

MBL	Mannose-binding lectin
MASP-1	Mannose-binding lectin-associated serine protease-1
GEO	Gene Expression Omnibus
RNA-seq	RNA sequencing
ssGSEA	Single-sample gene set enrichment analysis
GSVA	Gene set variation analysis
HLA	Human leukocyte antigen
IL	Infiltrating lymphocyte
CRP	C-reactive protein
PCT	Procalcitonin
PCA	Principal component analysis
APCs	Antigen-presenting cells
TNF	Tumor necrosis factor
PBMC	Peripheral blood mononuclear cells
APACHE II	Acute Physiology and Chronic Health Evaluation
RT-qPCR	Real-time quantitative polymerase chain reaction
ELISA	Enzyme linked immunosorbent assay
ROC	Receiver operating characteristic
AUC	Area under the curve
TBI	Traumatic brain injury
SOFA	Sequential organ failure assessment
DEGs	Differentially expressed genes
GO	Gene ontology
MF	Molecular function
BP	Biological process
CC	Cellular component
KEGG	Kyoto Encyclopedia of Genes and Genomes
TAFI	Thrombin-activatable fibrinolysis inhibitor
MV	Microvesicles

## Footnotes

^1^ http://www.ncbi.nlm.nih.gov/geo

^2^https://cibersortx.stanford.edu/

^3^ https://www.proteinatlas.org/
