# Supplementary figures and images for: Systematic analysis of MASP-1 serves as a novel immune-related biomarker in sepsis and trauma followed by preliminary experimental validation
Source: Front Med (Lausanne). 2024 Feb 7;11:1320811. doi: 10.3389/fmed.2024.1320811 (PMC10879275; doi:10.3389/fmed.2024.1320811)

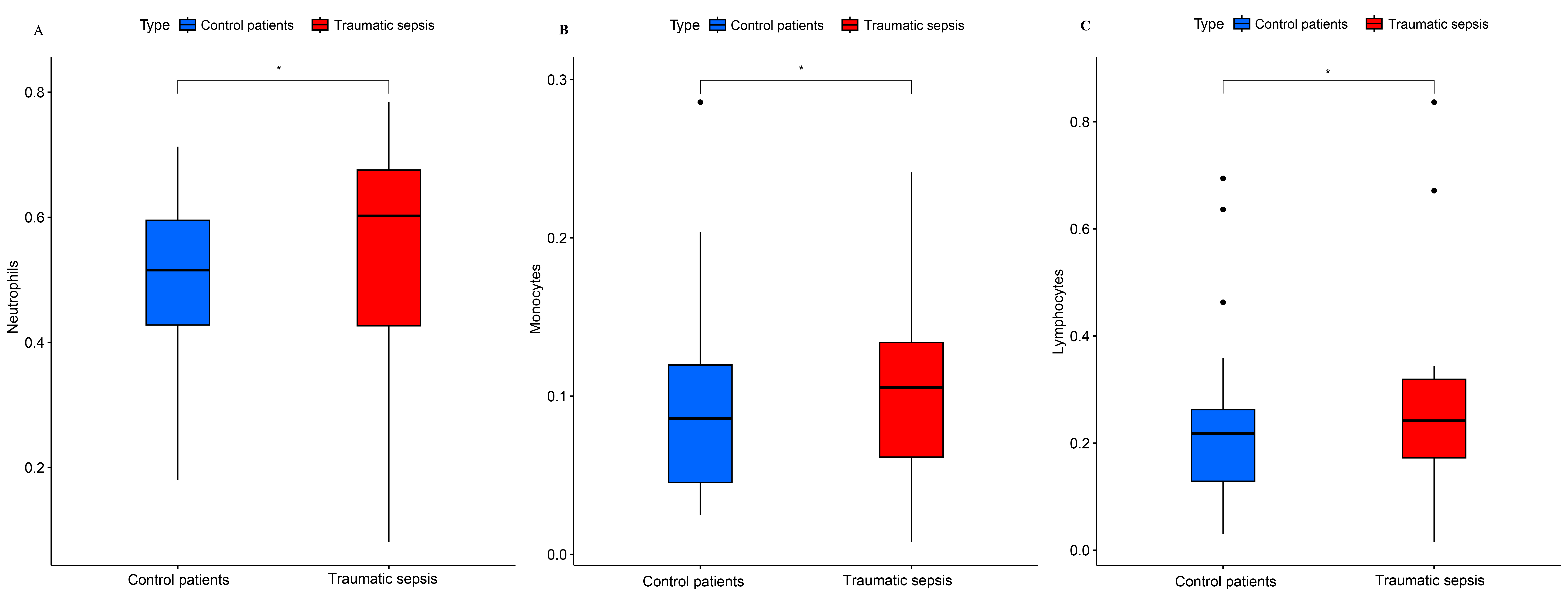

Supplement: Supplementary file 4 [file Image_1.TIF]

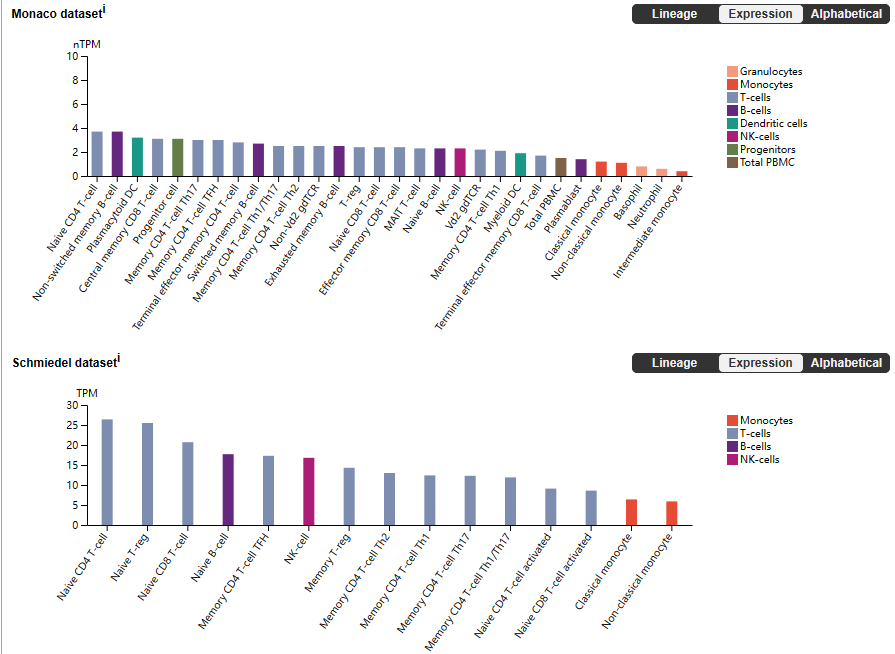

Supplement: Supplementary file 5 [file Image_2.PNG]
